# Children’s Understanding of Self-Focused Humor Styles

**DOI:** 10.5964/ejop.v12i3.1067

**Published:** 2016-08-19

**Authors:** Lucy Amelia James, Claire Louise Fox

**Affiliations:** a School of Psychology, Keele University, Keele, Staffordshire, United Kingdom; Department of Psychology, University of Western Ontario, London, Canada

**Keywords:** children, humor, humor styles, paired interviews, qualitative research

## Abstract

It has been proposed that four main styles of humor exist, two which are thought to be adaptive (affiliative, self-enhancing) and two which are thought to be maladaptive (aggressive, self-defeating). Whilst the existence of these four humor styles has been supported in older children, it is suggested that for younger children, self-enhancing and self-defeating humor may develop at a later point. To investigate this further, the current research involved five semi-structured paired interviews with children aged eight to eleven years to explore the use and understanding of self-enhancing and self-defeating humor in this age group. Findings indicated that use of both self-enhancing and self-defeating humor were apparent in some children, but not all. It therefore seems appropriate that attempts to investigate humor in this age group should aim to include all four styles of humor. The current research also demonstrated the value of paired interviews when carrying out this sort of research with children.

## Introduction

Martin, Puhlik-Doris, Larsen, Gray, and Weir (2003) proposed four types of humor which have been previously measured in adults using the humor styles questionnaire (HSQ). Whilst two of the humor styles, affiliative and self-enhancing, can be considered adaptive, the remaining humor styles, aggressive and self-defeating are considered to be maladaptive. Furthermore, the humor styles can also be categorised into self-focused and other-focused humor styles. Affiliative and aggressive humor can be considered as other-focused, whereas the focus of the current article, self-enhancing and self-defeating humor, are thought to be self-focused (Kuiper, Kirsh, & Leite, 2010). Whilst work exploring the role of these humor styles has been plentiful, research exploring their development in children has been limited.

The first of the four humor styles, affiliative humor was described by Martin et al. (2003) as using humor to amuse others and facilitate relationships, often through the use of jokes and funny stories. This friendly and non-hostile form of humor has been found to be positively related to variables such as self-esteem and well-being and negatively related to anxiety and depression (Chen & Martin, 2007; Kazarian & Martin, 2006; Kuiper, Grimshaw, Leite, & Kirsh, 2004; Martin et al., 2003). Aggressive humor on the other hand can be described as using humor at the expense of others, often without regard for their feelings. This can include the use of teasing and sarcasm. This less benign form of humor has been found to be related to hostility and aggression (Chen & Martin, 2007; Martin et al., 2003).

Turning to the self-focused humor styles, self-enhancing humor is thought to be closely related to the concept of coping humor and is consistent with the Freudian definition of humor as a healthy defence mechanism (Freud, 1928). It is described by Martin et al. (2003) as the type of humor used to maintain a positive outlook on life, to boost the mood or to deal with difficult situations. As stated by Martin, Kuiper, Olinger, and Dance (1993) having a humorous perspective can help people to distance themselves from their problems which could make them appear less threatening and help them to view difficult situations in a more objective way. This style of humor was again found to be positively related to variables such as self-esteem and well-being and negatively related to anxiety and depression (Chen & Martin, 2007; Kazarian & Martin, 2006; Kuiper et al., 2004; Martin et al., 2003). Moreover, in terms of social relationships, users of self-enhancing humor may appear confident and self-assured which could ultimately increase others’ positive opinions of them (Klein & Kuiper, 2006). As found by Kuiper and Leite (2010), self-enhancing humor was associated with higher ratings for socially desirable personality attributes compared to the maladaptive humor styles.

Self-defeating humor involves an individual’s attempts to use humor at their own expense often by putting themselves down or letting themselves be the butt of others’ jokes. As Martin et al. (2003) highlighted, self-defeating humor refers to excessive self- disparagement often to ingratiate oneself or to gain acceptance from others. Whilst this type of humor may appear amusing, it can reflect an inner neediness or lack of self-confidence. For example, in children it may be used by class clowns, victims of bullying and those of low social status (Fabrizi & Pollio, 1987; Klein & Kuiper, 2006). As found by Kuiper and Leite (2010), self-defeating humor can have serious detrimental effects on the impressions formed by others. It often involves both belittlement of the self and also the repression of an individual’s emotional needs, and so self-defeating humor may be particularly damaging (Martin et al., 2003). Hence, this style of humor has been found to be negatively related to well-being and self-esteem and positively related to anxiety and depression (Chen & Martin, 2007; Kuiper et al., 2004; Martin et al., 2003; Saroglou & Scariot, 2002). As discussed by Fox, Dean, & Lyford (2013) self-defeating humor may reinforce negative self-beliefs which could perpetuate these symptoms further.

A number of studies have highlighted relationships between children’s humor and their psychosocial adjustment. For example, Sherman (1988) found that children rated by their peers as humorous were also rated as less socially distant; whilst Masten (1986) found that better humor production and comprehension were associated with better social competence. Similarly, Freiheit, Overholser, and Lehnert (1998) found a negative relationship between humor and depression in adolescents. Klein and Kuiper (2006) stated however, that although these findings are encouraging, they are based on the assumption that humor is predominantly positive. They argued that Martin et al.’s (2003) humor styles model should be extended to consider the positive and negative aspects of humor during childhood and particularly the impact on children’s social relationships. In response to this, Fox et al. (2013) successfully adapted the humor styles questionnaire for children, finding a number of associations between humor and psychosocial adjustment. For example, self-defeating humor was found to be negatively related to social competence and self-worth and positively related to anxiety and depression. Whilst the measure was found to be suitable for secondary age children above the age of eleven, it was not found to be suitable for children below the age of eleven. Fox et al. (2013) therefore suggested that attempts to measure humor in younger children should initially include just affiliative and aggressive humor. They argued that due to their reliance on cognitive processes, self-enhancing and self-defeating humor may develop at a later point. Moreover, younger children may not be fully aware of their own use of humor.

It is suggested however, that children’s potential to use all four types of humor should not be ignored, particularly as evidence of coping humor has been found in younger children (Dowling, 2014; Führ, 2001). Furthermore, it has long been proposed that children use humor for emotional mastery and to deal with many of the challenges of socialisation (McGhee, 1979). Similarly, Altshuler and Ruble (1989) showed age related increases in children’s ability to manage emotions using more cognitive strategies. They argued that younger children can recognise that thoughts can be manipulated and that with age, children learn how to use strategies to effectively manage their emotions. Additionally, in terms of self-defeating humor, children have also been found to display self-derogatory attributions which have been linked with depressive symptoms (Nolen-Hoeksema, Girgus, & Seligman, 1991). When children begin to externalise these thoughts to make others laugh however is a question which remains unanswered.

Dowling (2014) used focus groups with children aged seven to twelve years to investigate understanding of humor. She found that children used humor to deal with a range of stressors associated with personal relationships, school and home life and were able to give examples to support the benefits of using humor. Dowling (2014) also argued that researchers make assumptions about children’s experiences of humor and that understanding humor from a child’s point of view is necessary to offer insight into its use as a coping strategy. Führ (2001) conducted interviews with children aged six to twelve years to investigate humor as a coping tool. It was found that whilst children indicated that humor is a useful coping strategy for dealing with problems or events, it is not helpful when dealing with serious issues or deep-felt emotions.

The current work therefore adopted a qualitative approach as used by Führ (2001) and Dowling (2014), to explore the potential use of self-enhancing and self-defeating humor in primary aged children and also children’s understanding of these humor styles. Whilst previous attempts to investigate children’s humor have included the use of interviews and focus groups, paired interviews have not been extensively utilised. As discussed by Highet (2003) paired interviewing is a fairly novel approach to interviewing young people. This approach, however, is thought to have a number of benefits including obtaining a better balance between the interviewer and participants, building trust and rapport, and creating a comfortable, supportive setting (Highet, 2003; Mauthner, 1997; Mayall, 2000). Moreover, interaction between participants may also be valuable (Arksey & Knight, 1999). If the interviews suggest that children are able to both use and understand self-focused humor styles, it should be concluded that all four humor styles should be examined in children between the ages of eight and eleven.

## Method

### Participants

Ten children who were members of the school council at a UK primary school were recruited. The participants were aged 8-11 years and in school years 4, 5 and 6 with a mean age of 9.60 years (SD = 1.08) consisting of seven girls and three boys. Parental consent was gained using the opt-in method, whereby a letter detailing the research was sent to parents and guardians a week prior to the interviews. They were then required to return an attached consent form to give permission for their child to participate. The process and nature of the research was explained to the children and they were asked if they would like to take part. They were assured that participation was not compulsory.

The interviewees were as follows: Interview A – a male and female, Emily and Tim, both aged 10. Interview B – two females, Natalie and Bella, both aged 11. Interview C – two females, Samantha and Rachel, aged 9 and 10. Interview D – a male and female, Catherine and Jay, aged 9 and 10. Interview E – a male and female, Gregory and Laura, both aged 9. NB: All names have been changed.

### Materials

Twelve interview questions suitable for upper primary aged children were developed to investigate self-enhancing and self-defeating humor. To assess the suitability of the questions for this age group, the Flesch reading ease score and Flesch-Kincaid grade level of the interview questions were examined (Flesch reading ease score of the questions = 81.1, US grade level = 5.6). This suggested that the questions would be suitable for children in year 6 (aged 10-11). As the questions would be read aloud to the children, they were deemed to also be appropriate for those in years 4 and 5. Six questions asked about self-enhancing humor and six asked about self-defeating humor. For example, for self-enhancing humor, the children were asked questions such as ‘When you are feeling sad, do you ever think of something funny to cheer yourself up?’ For self-defeating humor, an example question was, ‘Do you think letting others laugh at you is a good way to make friends? The questions were developed based on both the child humor styles questionnaire (Fox et al., 2013) and the adult humor styles questionnaire (Martin et al., 2003). In the case of a child answering only “yes” to a question, indicating use of self-enhancing or self-defeating humor, to elicit more detail they were then prompted to expand or discuss a time when they used that form of humor. For example, they were asked ‘can you tell me a bit more about a time when you did that?’ or ‘can you tell me a little more about what happened?’ When children responded to a question with “no”, it was indicated that their answer was adequate by moving onto another question.

### Procedure

A local primary school head teacher was approached by email and agreement to participate was received. Due to the need for interviews to be recorded and to be conducted outside of the classroom a parental opt-in method was used. Interviews were conducted after school during school council sessions to avoid interrupting lessons. To allow the children to talk freely, participants met with the researcher in the school meeting room.

Prior to the paired interviews, a standardised preamble was used to ensure that information was delivered to children consistently. It was stressed to the children that participation was their choice and that it was acceptable for them to withdraw at any point. To encourage the children to talk honestly, the recording device was pointed out and it was emphasised that the recordings would not be heard by anyone else. The children were however made aware that a teacher might be informed in the case of them mentioning something that raised concerns regarding safety. Taking into account the presence of two children, it was also suggested that children should not discuss the interviews with other children to ensure confidentiality. Following the end of the interview, the children were fully debriefed.

#### Data Analysis Procedure

After the recordings had been transcribed verbatim, the transcripts were analysed using thematic analysis using the process outlined by Braun and Clarke (2006). Firstly, they were read through a number of times and brief notes were made surrounding the parts that initially seemed of particular interest to the topic. Following on from this, particular relevant words and phrases were highlighted, drawn out and treated as initial codes. These initial codes were then interpreted further with similar phrases collated together to aid the search for reoccurring themes. After themes had been identified they were reviewed. This process involved considering whether themes needed to be broken down into subthemes and checking that all themes were supported by enough data. Subsequently, suitable names for the themes were decided upon. As described by Shenton (2004), a number of provisions were made to address Guba’s (1981) criteria for trustworthiness in qualitative research. In terms of data analysis, this was carried out with the assistance of a colleague with previous experience of qualitative methods. To reduce bias, based on the researcher’s previous knowledge of the four humour styles, the colleague had no prior knowledge. During the analysis process it was ensured that the final themes represented the majority of the data, a previously identified theme relating to using humor to disguise feelings was abandoned due to having less data to support it.

## Results

Through the completion of thematic analysis, three main themes were identified, ‘humor for the benefit of the self’, ‘laughing alone’, and ‘humor at the expense of the self’. The themes are explored in the order in which they became apparent from the transcripts.

### Humor for the Benefit of the Self

On a number of occasions throughout the interviews, several participants referred to humor being able to change their feelings for the better. This often occurred when children were asked if being a funny person stopped them from being sad, “I don’t want to be a sad person, if you think on the bright side and think of something funny it usually cheers you up. You want to be cheered up you don’t want to be dull and sad” (Interview D, Jay). It seems here that Jay believes that thinking of something funny is an important part of being cheered up and part of the process of being able to “think on the bright side”. Use of the word “usually” also suggests it is something he does regularly that often works. This was again demonstrated by another child when the same question was asked, “Because it could easily change your emotions. Instead of being sad you’ll also feel like you’re lonely and desperate but when like you’re a funny person, you can always think of something that’s funny” (Interview A, Tim). Here, the ease with which thinking of something funny can change the way you feel is emphasised. However, it may be that the child is drawing some kind of distinction by referring specifically to “a funny person” perhaps implying that this is a strategy that is not used by everybody.

When some children were asked if humor helps them to cope with situations that are hard or difficult, they were able to refer to particular circumstances where humor could be used. For example, one child mentions having to say goodbye to someone, “Probably when you have to say goodbye to somebody, so you try to think of the good things and funny things for when you’re next going to see them and the good times and the funny times you’ll have” (Interview C, Samantha). Not only is the child here acknowledging that humor could be used in a difficult situation like having to say goodbye to someone, but they also seem to know how humor could alleviate particular feelings. It seems they are able to envisage how humor could change their experience of the situation, specifically by thinking back to funny times and imagining those they will have in the future. Other children also referred to specific situations in which humor would, or has, been used as a sort of coping strategy, “Yeah, I think of like if everyone’s gone against me like Catherine said, I would think of the times when it was funny. Like the time in the hall with that yogurt, it went a bit wrong” (Interview D, Jay). Here, as the child refers to others going against him, he seems to be referring to conflict with his peers. It may be that humor helps him to feel better about the situation, but humor in this instance may also help the child to deal with or confront the situation perhaps by appearing to others as if he is coping.

As well as using humor to deal with emotions, a child also referred to an incident in which thinking of something funny helped to alleviate physical pain. “Erm, we were playing and someone tugged me and it really hurt, so I just thought of something funny and it didn’t hurt anymore.” (Interview C, Samantha). The specific manner in which humor functions as a coping tool needs to be considered. For example, it may be suggested that humorous thoughts can mask negative ones or allow them to be thought about in a more positive way. However, in the case of dealing with physical pain it would seem its role may be different. For instance, perhaps humor here is purely acting as a distraction to the pain that is being felt.

Whilst many of the children agreed that humor can help, some children did disagree. This seems to indicate the presence of individual differences. For example, this was evident for one child when asked if thinking of something funny can help when they are feeling worried. “Most of the time if it’s really serious probably not because that might get you thinking, oh this is funny so that you don’t need to think this is too important” (Interview E, Laura). Here Laura indicates that she believes humor might have a negative role, perhaps in preventing a situation from being approached as it should be. This could indicate that potentially, she does not understand that using humor may be beneficial in facing problems. However, in referring specifically to serious problems, it is not clear what her thoughts may be for less serious problems. Perhaps by thinking about situations where humor may or may not be an effective strategy, Laura is showing a good understanding of this style of humor.

Similarly, another child also revealed that she doesn’t believe that thinking of funny things would help to cheer her up: “I can’t really make myself happy, I just usually think of sad things and then I get even more sadder” (Interview D, Katherine). This may again represent individual differences and also that the child doesn’t really understand or know how humor can be used to improve her mood. She may therefore be lacking in the necessary skills to use humor in this way, skills which if acquired could prove valuable.

### Laughing Alone

At many points throughout the interviews, it became clear that children believe that humor is something that can still be used or experienced when they are alone, “We had a test today and I finished and everyone else was like checking their work and I’d already done that so I just thought of funny things” (Interview C, Samantha). It seems that humor in this case is something that has been created in the mind probably as an entertaining way to fill the time.

Most notably, children don’t just see humor as something that occurs in a social context with the need for others to be involved. It also seems that thinking back to occasions where something humorous has occurred is something children do when alone, “When I’m alone in my bedroom I normally, if I get bored and I’m doing homework I normally think back to the work we did in the classroom and then if something funny went on I’ll laugh about that” (Interview A, Emily).

Again, humor is occurring when the child is alone, but involves a process of reflecting back, something which could also be used as a coping method. Another instance where a child refers to thinking back involves him talking about his mum, “I would think back to my mum and think that she always says something funny to me so I would think of mum and she would say something funny and I’ll laugh” (Interview A, Tim). Thinking about a specific person who makes them laugh and about what they would say doesn’t involve the need for others. It can also be argued that for Tim spending time with his mum, who seems to use humor frequently, may have helped him to adopt positive uses of humor. For instance, Tim may also have learnt to try and use it regularly even when others are not present.

Finally, when asked if they still find things to laugh at when they are on their own, one child suggests that the things they laugh at when alone are different than when they are with other people, “I do something funny that I wouldn’t do if other people were watching if it’s a bit weird or embarrassing” (Interview E, Laura). This suggests that careful thought may go into the humor that children use when in the company of others. This may be positive in that children take fewer risks regarding what is appropriate and are aware that boundaries of what is acceptable exist. On the other hand, being over conscious could be restrictive. In general laughing when alone seems to offer children the chance to enjoy their own humor without the concerns of being judged by others, and perhaps also further develop their humor skills.

### Humor at the Expense of the Self

When children were asked if they let people laugh at them more than they should, it seemed they were recalling specific incidents in their minds when they had done this. For example, one child referred to others making fun of her appearance, “Like people laugh at me for being so tall and it really bugs me sometimes” (Interview B, Bella). It is proposed that although the topic being discussed was ‘letting others laugh’ it could be very difficult for the child to confront the laughing children and explain her feelings regarding being the butt of their jokes. This seems like a key example of a child experiencing being on the receiving end of aggressive humor and the negative impact it can have. It is not however clear whether the child may have used her height previously with the intention of making others laugh. Neither can it be known whether it is something she might consider in the future.

It does seem apparent in some cases, that children may let others laugh at them intentionally; for example, as shown when asked if they ever talk about things they are not very good at in a funny way, “Yes because they might think that you trust them to keep it, like because it might be a bit personal. So you could feel like you could trust that person and they might feel that too” (Interview A, Emily). Although the child agrees with the question, they believe this may actually help to build their relationship through demonstrating trust. Whilst the child seems to believe this is a useful strategy, it can be argued that negative consequences are possible. In this case, the child may become more focused on the things they are not very good at, which in turn may have a negative effect on their self-esteem. Similarly, in response to the same question, another child also talks about letting others laugh about something she is not very good at, “I agree but sometimes it’s nice like I don’t really like gymnastics and I’m not good at it so we always sometimes have a little joke about that” (Interview C, Samantha). While it may be the case that it is healthy and constructive for her to joke with her friends about gymnastics, there may be the potential for detrimental consequences in similar situations, particularly if the joking were to become excessive. For instance, other children may not understand or respond the same way and feel they need to discourage them. The child seems to be unaware of these potential responses. In addition, it is perhaps debatable whether young children actually possess the skills to successfully make jokes at their own expense. It is also possible that others may in the future use the things they have laughed about to make jokes or tease, perhaps in front of others. This may be particularly worrying as the outcome of regular teasing may in some cases be similar to those of bullying.

When asked if making fun of yourself makes others laugh, one child suggests that it doesn’t. However, when referring to the reactions of others, one child suggests that making others laugh by making fun of herself is something that she has done, “No, because they always say ‘oh that’s not true’” (Interview A, Emily). Gaining this sort of reaction from people may have resulted in the child refraining from making fun of themself in front of others and some awareness of the negative consequences may have been acquired. This can be regarded positively in that a humor style considered to be maladaptive might no longer be being used. Nevertheless, in using this form of humor, the child may have inadvertently portrayed an inner neediness or caused others to tire of reassuring them, possibly putting strain on their relationships. In not gaining the desired response of others laughing, this may also have deterred the child from attempting to use humor in a more positive way in the future. In addition, it seems important to consider whether the other children genuinely don’t find the self-defeating humor funny, or whether they just feel it is their role to build their friend’s confidence.

As demonstrated above, during the interviews some children did agree that they use or have used humor at the expense of themselves. Comments were also made however which suggest that some children don’t use this form of humor, and are perhaps confused as to why it would be used, “Well I think you really shouldn’t do that because that’s not really true friendship and it’s nice to have a laugh but you shouldn’t have to laugh at yourself” (Interview C, Rachel). This implies that the child believes that making fun of yourself is wrong which seems positive in indicating avoidance of negative humor styles. It could also represent that some children may not fully understand the concept of self-defeating humor or why anyone would purposely make fun of themself. This may also be indicated by a further comment, “No because they’re laughing at you. It’s fine to laugh with people but laughing at yourself is really not nice at all” (Interview C, Rachel). It seems that some children are able to draw a distinction between being laughed at and laughing with others. They put particular emphasis on the unpleasantness of being laughed at which again could point towards confusion as to why this style of humor would ever be adopted.

Additionally, some children seemed to consider the humor they use when meeting or making friends with new children, “If a kid comes in the class you don’t want to embarrass yourself because then they’d think of you as the class clown maybe and might like you even less” (Interview D, Jay). In this case, Jay seemed to reveal that they think letting others laugh at you can be bad for your reputation or the impression you make on others. Whilst this may be a well-adjusted child, cautious of the humor they use, it could also indicate a lack of awareness for the form of humor.

The child did, however, go on to reveal when asked if making fun of themself makes people laugh, that they are aware that other people do purposely make fun of themselves to create humor, “[because] lots of people, they become comedians by doing that” (Interview D, Jay). A number of things can be noted about the statement. Firstly, the child refers to this type of humor being used in terms of comedians, a group of people known specifically for the humor they use and who are also adults. Children may therefore associate this type of humor with adults and not necessarily with children. In addition, using this type of humor as a performance or to entertain may be considered very differently to using this type of humor in a typical personal or social situation.

## Discussion

To our knowledge, this is first study to adopt a qualitative approach to explore children’s use of the humor styles proposed by Martin et al. (2003). Although work has been carried out to investigate the links between children’s humor styles and adjustment, less is known about the way children actually use and understand different styles of humor. Moreover, whilst children’s use of aggressive and affiliative humor may be highly visible, their use of the self-focused humor styles may be harder to detect.

In summary, the majority of children seemed to have an understanding of the concept of self-enhancing humor, as well as indicating that it is a form of humor that they use, predominantly to make themselves feel better. A small number of children did however seem to show less understanding of this humor style as well as indicating that they probably would not use it. This seems to highlight the existence of individual differences in using humor to cope. Conversely, children unanimously agreed that they can still laugh about things when alone as opposed to with others, representing their belief that humor is not just something that can be used socially. Individual differences were more notable for self-defeating humor. Some children indicated that they have used this form of humor, but seemed unaware of the negative consequences its use may result in. Others implied that they wouldn’t use it at all and seemed to show confusion towards why humor would be used in this way. Asking children more specifically about the potential consequences of using different styles of humor may therefore be beneficial in future research.

Using a qualitative approach offered important insight into the ways that different humor styles are understood and used. Considering self-enhancing humor, as found by Führ (2002), it seemed evident that some children firmly believed that humor can change emotions and that it can be particularly useful in preventing or alleviating negative emotions. This also supports previous research that implies that children are aware that thoughts can be manipulated (Altshuler & Ruble, 1989). This seems to be strong evidence to back up the notion that humor can act as an important coping tool even in children younger than eleven years. It could also be used to challenge the views of those such as Høffing (1916) who believed that children are not yet mature enough to use humor as an attitude towards life.

Children also referred throughout to incidences in which they used humor when alone without the presence of others, often through reflecting back. This non-social use of humor again seems to be a probable indication that for younger children, as well as older, humor can be used for the benefit of the self. Being able to use humor when alone, it seems, may provide children with an enjoyable opportunity to use humor which they may feel uncomfortable sharing with others. It may also be a way for children to develop their humor skills for example, by encouraging them to consider what is appropriate, especially when meeting new people. Creating the wrong impression through using too much or the wrong kinds of humor early on seemed concerning to the children. Furthermore, it appears possible that by being raised or surrounded by others, perhaps parents, who regularly use or introduce humor at times of upset, may lead to children acquiring these strategies for themselves. Dowling (2014) also suggested that family may influence children’s humor skills. Thus, creating awareness of the different forms of humor in caregivers may be worthwhile in the future.

It is acknowledged that children could have agreed with the interview questions to please or because they felt uncomfortable disagreeing, particularly if the other child had answered differently. However, it was apparent that children could refer to specific situations in which they might be able to use humor to make themselves feel better. As Dowling (2014) stated, research was needed to investigate under what circumstances children might use humor to cope. This insight could be particularly useful when contemplating how to approach raising awareness of the benefits of adaptive humor styles with young children. Also, children seemed aware of the way in which humor could help, for example, being able to look at a situation, such as saying goodbye to someone, in a more positive light, can help individuals to approach events differently (Martin, Kuiper, Olinger, & Dance, 1993). Evidence of humor masking negative feelings seemed to be noticeable although it could also be that humor has the potential to function as an effective distraction technique. This became apparent when humor was discussed in relation to its role in reducing physical pain. This evidence seems supportive of claims made by Goodenough and Ford (2005) who suggested that for hospitalised children employing distraction strategies; humor could be used as an effective way of dealing with pain related distress.

It also seems relevant to question whether children specifically decide to think of funny things. Whilst use of humor as a coping mechanism could be an automatic reaction or trait, it could also be a deliberate strategy or a skill which has been learnt (Martin, 1998). As stated by Dowling (2014), work is still needed to investigate the deliberate choice of humor as a coping technique. For children who didn’t agree that humor can be used as a coping tool, they may not have acquired this skill or strategy. For example, one child said that they can’t use humor to make themselves happy. Whilst this lack of ability to use humor could be a reflection of the child’s mood, it could also represent a lack of awareness of the potential positive outcomes of humor. Another child however suggested that humor should not be used when problems are serious. This supports the findings of Führ (2001) who found that children believed that humor could be useful only when dealing with less serious issues. In relation to this, Lefcourt, Davidson, Prkachin, and Mills (1997) proposed two forms of coping strategies that may be related to humor. Firstly, emotion focussed coping – finding humor in a difficult situation to reduce negative reactions and secondly problem-focussed coping – using humor to alter a stressful situation (Abel, 2002). For some situations, whilst taking a problem-focussed approach to using humor may be useful, for other issues, where the situation cannot be changed, it may be less appropriate. Using emotion-focussed humor coping, for example using humor as a distraction mechanism may still be beneficial however. In the same way, for situations that need to be addressed, emotion-focused humor coping might not be appropriate.

Turning to self-defeating humor, individual differences in both use and understanding were more pronounced. Some children indicated that they would be unlikely to use self-defeating forms of humor and appeared to show confusion as to why it would be used. For example, they referred to the difference between being laughed at and laughing with others. In terms of understanding however, they were able to refer to its use by comedians, showing an awareness of its existence. As discussed, this may suggest it is a style of humor they may associate more with adults, and with those for which creating humor is a profession. Children may therefore see this as more acceptable compared to its use by peers. For example, Klein and Kuiper (2006) suggested that self-defeating humor is used by class clowns. Whilst these children may appear amusing, their use of humor often reflects an inner neediness which may be seen as less acceptable.

Whilst it was assumed by some children that no harm would come from making jokes at their own expense, it is suggested that frequent use of these sorts of jokes, may have potentially harmful consequences that children may be unaware of. For example, as Fox et al. (2013) discussed, through regularly referring to their shortcomings, these beliefs could be reinforced which may in turn have a negative impact on their self-esteem. As Martin et al. (2003) pointed out, self-defeating humor refers to excessive self-disparaging humor with a key element being belittlement of the self.

One response from a child alluded to reactions from peers who dismissed the self-defeating comments, possibly to provide reassurance. This kind of reaction however, could also hint that children may be put off by the lack of confidence or neediness that is portrayed, which could in turn lead to rejection or weak relationships. The interviews indicated however, that some children may believe that sharing this sort of humor with others is an effective way to build close relationships. As previously mentioned, children may not possess the skills to use humor at their own expense. They may also be unaware of the point at which self-depreciating humor becomes excessive, for example when laughing with others about things they are not good at. Through not receiving the desired reaction from peers in response to use of humor at their own expense, the risk is that these children may be discouraged from attempting to use other, more positive forms of humor in the presence of others. Ultimately, if children lack understanding and are unaware of the potentially negative consequences of self-defeating humor, it seems important to find ways to raise their awareness.

It also seems apparent that in some cases children let others laugh at them, not through choice but because they do not know how to make it stop, highlighting the unpleasantness of being on the receiving end of aggressive humor, as discussed by Shapiro, Baumeister, and Kessler (1991). This could perhaps have been delved into further; however, using paired interviews may have made it more difficult for some children to expand due to the presence of another child. Interviewing children in pairs did however have a number of benefits. For example, the children appeared relaxed and were able to interact freely and bounce ideas off each other (Arksey & Knight, 1999).

There are a number of limitations to the current research which should be acknowledged. Firstly, the small sample size which consisted of only school council members. It is possible that these children may be well adjusted and therefore more likely to use self-enhancing as opposed to self-defeating humor. More evidence of self-defeating humor might have been apparent if the sample was more diverse, whilst self-enhancing humor may have been less evident. Moreover, the study took place in only one school and therefore one location, meaning it would be premature to conclude that the findings can be generalised. However, it must be acknowledged that this should never be a goal of qualitative research. Lastly, the study aimed to explore both children’s use and understanding of the self-focused humor styles. The interview questions however only directly asked participants about their use of humor. Asking more specifically about the consequences of humor may allow for a greater level of understanding.

Overall the current study provided understanding and seemed to indicate use of both self-enhancing and self-defeating humor in some children, but not all. This highlights the need to raise children’s awareness of the existence of adaptive and maladaptive styles of humor. In addition to this, the questions used during the interviews appeared to be well understood by the children. This being the case, it was concluded that the same questions should be adapted in questionnaire based research to be carried out with larger and more diverse samples. This would then allow for the examination of differences based on gender, age and culture to also be explored. Ultimately, further research examining children’s humor styles should aim to utilise qualitative methods alongside the more traditional quantitative approaches that are typically used in this field.
